# Okay to Stay? A new plan to help people with long-term conditions remain in their own homes

**DOI:** 10.1017/S1463423618000786

**Published:** 2018-11-15

**Authors:** Helen Chapman, Lisa Farndon, Rebekah Matthews, John Stephenson

**Affiliations:** 1 Head of Integrated Community Care, Combined Community & Acute (CCA) Group, Sheffield Teaching Hospital NHS Foundation Trust, Vickers Front Hall, Northern General Hospital, Sheffield, UK; 2 Clinical Research Podiatrist/Research Lead, Integrated Community Care and Primary Care and Interface Services Directorates, Sheffield Teaching Hospitals NHS Foundation Trust, Sheffield, UK; 3 Integrated Pathway Manager, Integrated Community Care Directorate, Sheffield Teaching Hospitals NHS Foundation Trust, Sheffield, UK; 4 Senior Lecturer in Biomedical Statistics, School of Human and Health Sciences, University of Huddersfield, Huddersfield, UK

**Keywords:** frail, elderly, hospital admission avoidance, long-term conditions

## Abstract

**Aims:**

To assess the ‘Okay to Stay’ plan to investigate if this reduces visits to emergency departments, unplanned admissions and elective admission to hospital in elderly patients with long-term health conditions.

**Background:**

The incidence of long-term conditions is rising as the elderly population increases, resulting in more people from this group attending emergency departments and being admitted to hospital. Okay to Stay is a simple plan for people with long-term conditions to help them remain in their own home if they suffer an acute exacerbation in their health. It was co-designed with professional and patient representatives with the aim of empowering patients and their carers to more effectively manage their long-term conditions.

**Methods:**

Data from 50 patients (20 males, 30 females, mean baseline age 77.5 years) were compared 12 months before implementation of the plan and in the subsequent 12 months, with the significance of effects assessed at the 5 per cent significance level using *t*-tests.

**Findings:**

Visits to emergency departments were reduced by 1.86; unplanned emergency admissions were reduced by 1.28 and planned elective admissions were raised by 0.22 admissions per annum. The reduction in visits to the emergency department was significant (*p* = 0.009) and the reduction in emergency admissions was significant (*p* = 0.015). The change in elective admissions was not significant (*p* = 0.855). The Okay to Stay plan is effective in reducing visits to the emergency department and unplanned hospital admissions in people with long-term conditions. This is a positive step to supporting vulnerable and complex patients who are cared for at home, and facilitates the recognition by the individual of the possibility to stay at home with the support of health professionals. There are potential cost benefits to the investment of initiating an Okay to Stay plan through the avoidance of visits to the emergency department and non-elective admissions to hospital.

## Background

Fifteen million people in England suffer from long-term conditions, and this group accounts for 70 per cent of all bed days and acute and primary care budgets (Department of Health, 2016). Age and long-term conditions are associated with higher readmission rates to hospital; readmission rates are 10 per cent in those aged 16–74 years but rise to 15 per cent in people over 75 years (Lyndon *et al*., 2014). Older people also have a longer length of stay than younger patients once admitted to hospital (Sager *et al*., 1996).

The incidence of long-term conditions increases with age, as does frailty; it is estimated that between a quarter and half of people older than 85 years are estimated to be frail (Collard *et al*., 2012). Frailty has been defined simply as a state of increased vulnerability to adverse outcomes (Frieswijk *et al*., 2004) or more complexly as: ‘a condition or syndrome that results from a multisystem reduction in reserve capacity, to the extent that a number of physiological systems are close to, or pass, the threshold of symptomatic clinical failure’ (Campbell, 1997).

There is still a paucity of evidence to show the effectiveness of hospital avoidance programmes. A number of different initiatives have been trialled to reduce length of hospital stay and readmission rates; including tailored structured discharge plans, which may have a small effect, but overall cost benefits are still inconclusive (Shepperd *et al*., 2010). A randomised controlled trial comparing a community in-reach rehabilitation and care transition with the ‘usual’ discharge to rehabilitation service on length of hospital stay and readmission rates found no difference between the two approaches (Sahota *et al*., 2016).

Virtual wards have also been introduced. These can include coordinated care using a combination of telephone calls, home visits or clinic visits from a multidisciplinary team for several weeks after hospital discharge. When a virtual ward package of care post-discharge was compared with ‘usual care’ in a large multicentre Canadian study of nearly 2000 patients, no statistical difference was found between the two approaches in hospital readmission rates up to 12 months afterwards (Dhalla *et al*., 2014). Two hospital avoidance schemes using integrated care teams and approaches were compared with a control group; again no difference was found in rates of hospital use after the intervention (Steventon *et al*., 2012). It is therefore important that any programmes must target people at risk of readmission; the intervention must be cost-effective and clearly show that it does reduce hospital admission rates.

**Figure 1:**
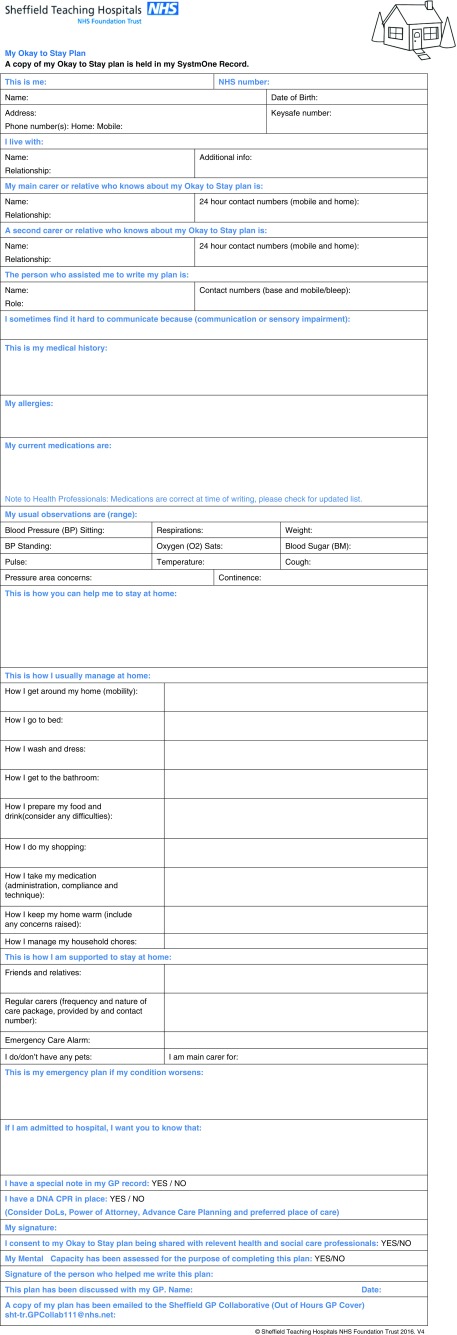

Okay to Stay is a plan for mainly frail elderly people with long-term conditions to help manage acute exacerbations in their health at home (Figure 1). It was designed by members of the Integrated Care Team at Sheffield Teaching Hospitals NHS Foundation Trust (STHFT) in close collaboration with stakeholders including the Sheffield Citizens Reference Group. This project was introduced as part of a shift towards more integrated care for people with long-term conditions. This has been recognised as an important factor including the identification of patients with long-term conditions; providing holistic, person-centred health and social care services, and encouraging patients to help manage their own care (NHS England, 2018). Its aim is to reduce unnecessary hospital stays, visits to the emergency department and calls to the out-of-hours GP service. Once a plan has been formulated in close collaboration with the patient and relatives/carers, each patient is supported with regular visits by community matrons or other members of the integrated care team, in combination with weekly phone calls (if appropriate) to monitor and manage their health conditions.

Patients are identified for an Okay to Stay plan by the community matrons. They are generally frail, older adults with multiple long-term conditions, and are known to have numerous admissions and extended lengths of stay in hospital, because of their complex health conditions. The process of initiating the Okay to Stay plan developed through several iterations in the early stages, with the following process being established:

- Community Matrons support the patient to complete the plan (with family members or carers involved).
- The plan is completed and saved on the Electronic Patient Record (SystmOne).
- The plan is shared with and discussed with the GP.
- The plan is emailed to the GP Collaborative (out of hours GP service) using the established protocols.
- Several copies of the plan are printed for the patient and family members to keep, and put in a prominent place in the home.
- The Community Matron provides any indicated follow-up, such as referrals on or changes to care.
- The plan is reviewed every three months or when indicated by significant change.

Following initiation of the plan, should the patient become unwell and access urgent care, such as the out of hours GP or the ambulance service, the responding clinician can then access the Okay to Stay plan as part of their care provision. As the patient is encouraged to take ownership of the plan, they are expected to bring it to the attention of any visiting health care professionals who may not know them well.

A small pilot study using the model with 32 patients showed a 41 per cent reduction in the number of episodes of emergency admissions: from 67 episodes in the 12 months prior to implementation, to 9 episodes during the following 5 months. There was also a reduction in calls to the GP collaborative (out-of-hours service) from 31 to 5 calls. The plan was evaluated on a purposive sample of five patients and their carers where a semi-structured interview was carried out by phone or in person. This found that the plan was helpful to both patients and carers as it empowered them to manage their own health. It also equipped them with detailed information that was available to other health care professionals (such as paramedics or on-call GPs) to more adequately decide, in an acute exacerbation of a condition, whether hospital admission was really necessary.

The plan costs approximately £241 per year per patient to implement. This includes approximately 1 hour per plan, plus follow up time of one hour for liaison with other health professionals, plus an hour every further three months in a 12-month period. The cost was estimated by the trust finance department, including preparation, travel and non-pay time. This is in addition to the ongoing support of these patients, who are regularly visited by our community nursing services.

## Methods

As the pilot study indicated that Okay to Stay plans were effective in reducing attendance to A&E, emergency admission and calls to the out of hours GP service, the plans were introduced to a wider group of patients with one or more long-term condition, living in their own homes. Data were collected between 2015 and 2017 from the electronic patient record. All patients on whom data were collected were included in the study.

The sample was summarised descriptively with demographic data (gender, age, living status, pre-existing conditions, medications, risk of falls) recorded at baseline, along with the numbers of attendances the emergency department, unplanned emergency admissions and planned elective admissions recorded during the 12-month period of the implementation of the Okay to Stay plan; and in a corresponding 12-month period before implementation of the intervention. Differences between pre- and post-admission statistics were also calculated.

Paired samples *t*-tests were conducted on each of the three outcome measures, using data from patients who remained alive throughout both pre- and post-intervention periods. The outcomes representing visits to the emergency department and unplanned emergency admissions were considered to be primary outcomes of equal importance; with planned elective admissions considered to be a secondary outcome. To avoid spurious findings of significance due to inflated familywise error rates arising from multiple comparisons, a Bonferroni-adjusted significance level of 0.025 was assumed for the two primary outcomes of visits to the emergency department and unplanned emergency admissions. Ninety-five per cent confidence intervals for all effects were derived. Effect sizes for significant effects were assessed using Cohen’s *d* statistic.

To ensure that the sample was not biased by the exclusion of patients who died during the course of the study (and hence could not provide full information relating to attendance to A&E, emergency admission and calls to the out of hours GP service), characteristics of deceased patients were compared against those who remained alive using independent samples *t*-tests; with any imbalances accounted for. Sensitivity studies were also conducted with deceased patients included as an additional assessment of the effect of the inclusion of these patients on study findings.

Secondary analyses were also conducted on the data to assess the effect of demographic factors on outcome measures. Independent samples *t*-tests were used to compare groups on categorical factors (gender, living status, risk of falls status) with respect to all measured outcomes: correlational analysis was used to assess the relationship between numerical variables (age, number of medications) with respect to all measured outcomes.

## Results

Data were collected between from 50 patients (20 males, 30 females). Patients ranged in age from 42.8 to 97.8 years at baseline, with a mean age of 77.5 years (*SD* 11.5 years). Twenty patients (40.8 per cent of valid responses) lived with a spouse or other family member(s), or in sheltered accommodation; 29 patients (59.2 per cent of valid responses) lived on their own. The living arrangements of one patient were not recorded.

All patients had one or more reported long-term condition. Hypertension, Type 2 diabetes, chronic obstructive pulmonary disease and asthma were the most frequently reported. All patients were taking medications regularly for their conditions. Twenty-seven patients (56.3 per cent of valid responses) were assessed as being at risk of falls: 21 patients (43.7 per cent of valid responses) were assessed as being at low or no risk of falls. Two patients did not have a falls risk recorded.

Nineteen patients (43.2 per cent of valid responses) were receiving some sort of social care package during the study period and 25 patients (56.8 per cent of valid responses) were not. The status of six patients with respect to this factor was not reported.

During the year-long implementation of the programme, five patients died. No admission data during either the pre- or post-implementation period was obtained from one patient. Independent samples *t*-tests revealed that there were no significant differences in the frequencies of A&E attendance, emergency admission and calls to the out of hours GP service between patients who remained alive throughout the full study period and those who died within the study period; hence exclusion of patients who had died would not bias the sample. These patients were excluded from the subsequent main analysis, which was conducted on 44 patients.

The number of visits to the emergency department, emergency admissions and elective admissions pre- and post-implementation of the intervention are summarised in Table 1. Amongst patients who remained in the analysis, compared with the pre-intervention period, visits to the emergency department were reduced by 1.86 visits per annum on average; unplanned emergency admissions were reduced by 1.28 admissions per annum on average, and planned elective admissions were raised by 0.22 admissions per annum on average.

**Table 1:** The number of attendances to the emergency department, emergency admissions and elective admissions pre- and post-implementation of the Okay to Stay Plan

	Pre-implementation (*M* (*SD*))	Post-implementation (*M* (*SD*))
Visits to emergency department	3.25 (4.20)	1.30 (2.48)
Emergency admissions	2.64 (3.07)	1.30 (2.20)
Elective admissions	0.300 (0.795)	0.320 (0.771)

Paired samples *t*-tests conducted to assess the significance of these differences revealed that the reduction in visits to the emergency department was significant at the 5 per cent significance level (*p* = 0.009). A 95 per cent confidence interval for the reduction was given by (0.478, 3.23). The reduction in emergency admissions was revealed to be significant at the 5 per cent significance level (*p* = 0.015). A 95 per cent confidence interval for the reduction was given by (0.264, 2.29). Application of the Bonferroni correction did not alter the inference of significance of either outcome. Sensitivity studies conducted on all patients (including those who deceased) revealed negligible changes to confidence interval widths and no changes to any inferences of significance or otherwise of any findings.

The effect of the intervention was moderately large in magnitude with respect to both primary outcomes: Cohen’s *d* = 0.405 for the reduction in visits to the emergency department; Cohen’s *d* = 0.364 for the reduction in unplanned emergency admissions. The reduction in elective admissions was not significant at the 5 per cent significance level (*p* = 0.855). A 95 per cent confidence interval for the reduction was given by (−0.266, 0.222).

Secondary analysis conducted on patient-level variables revealed no evidence for any effect of any of the measured variables (age, gender, risk of falls, number of medications, family status) on any of the measured outcomes.

## Discussion

The Okay to Stay programme has demonstrated a significant reduction in numbers of annual visits to emergency departments, and in the number of unplanned emergency admissions, controlling for multiple comparisons, in a mainly elderly population with multiple co-morbidities at moderate risk of falls. There is no evidence that the programme affects the number of planned elective admissions which might be expected as the main emphasis is to reduce unplanned admissions.

As this study was formulated as a paired design with no control group, it is not possible to definitively claim that the recorded reductions were due to the implementation of the intervention and not to some concurrent effects. However, there is no evidence for any concurrent systematic changes in the health or lifestyle of the patients, and the level of significance and substantive magnitude of the improvements observed are suggestive that the Okay to Stay programme has been successful in its aims. The secondary analyses conducted on patient-level variables revealed no evidence that changes in outcomes could be ascribed to any measured variable other than participation in the Okay to Stay programme. Furthermore, in an elderly cohort, an additional year of age might be expected to result in a slight deterioration of health and consequently an increase in visits to the emergency department and unplanned emergency admissions: the observed improvements are observed in spite of this countering age effect.

The exclusion of patients who died during the post-intervention period is justified as these patients can be shown to be typical of the wider cohort. This was verified by comparison of all recorded health and demographic statistics in patients who survived for the duration of the study and those who did not survive: no substantive differences on any characteristic were observed. Furthermore, any patient who died during the pre-intervention period would have automatically been excluded from the study.

## Conclusion

Okay to Stay promotes patient independence and enhances person-centred care. It also allows a better understanding of individuals’ health and social needs by the multidisciplinary team providing their care and other agencies that may become involved during exacerbations in health, such as out-of-hours GP services and paramedics. A copy of the plan is sent to the out of hours GP centre, and an alert is set up on the community and inpatient electronic patient record, if a patient is admitted to hospital.

The project has been improved by including the multidisciplinary team in the completion of Okay to Stay, including community support workers, GPs and therapists. We are also looking into sharing the plan regionally to match the regional ambulance service. We are now looking at the National Frailty Index and risk stratification in one area of the city to help identify patients who may be in need of an Okay to Stay assessment. As it is targeted at those who are most likely to use out of hours services and have unplanned emergency admissions to hospital it has the potential for large cost savings for the healthcare economy.
